# COMPANION CARE ASSOCIATED WITH REDUCTION IN ADMISSIONS AND EMERGENCY DEPARTMENT USE AMONG OLDER ADULTS

**DOI:** 10.1093/geroni/igac059.2927

**Published:** 2022-12-20

**Authors:** Kelsey McNamara, Ellen Rudy, Anne Armao, Kerri Towsley, Samantha Lang, Carla Perissinotto

**Affiliations:** Papa Inc., Miami, Florida, United States; Papa Inc., Miami, Florida, United States; SummaCare, Akron, Ohio, United States; SummaCare, Akron, Ohio, United States; Weill Cornell Medicine, New York, New York, United States; UCSF, San Francisco, California, United States

## Abstract

Older adults with unmet social needs, including social isolation and loneliness, have higher rates of hospital readmission and emergency department (ED) use. Our objective was to determine if Papa Inc., a nationwide service that pairs older adults with “Papa Pals” for companionship and assistance, was associated with reductions in readmissions and ED visits. SummaCare, a Medicare Advantage Organization in Ohio, partnered with Papa to offer free companion care to their members. We explored changes in inpatient admission rates and ED high-utilizers (defined as members with 4+ ED visits in a calendar year) from 2018 through 2021 using a claims-based analysis. Analysis included members who used 30+ minutes of Papa services in 2021 and had historical claims data from 2019 (n=1,420), matched 1:1 to a non-Papa comparison group (n=1,420) using a validated risk scoring model. Overall, average age was 78, 62% were female, 2% were ED high-utilizers in 2021. Increased enrollment in Papa occurred post hospitalization. The case-mix adjusted 30-day readmission rate in 2021 was 12.6% for Papa users (compared to 14.1% for non-Papa; and 14.5% for Papa users before enrollment), revealing a 1.5% to 2% decline in readmission rate after enrollment in Papa. Using case-mix adjusted relativities, Papa members had 0.89 readmissions for every 1.00 readmission for non-Papa users. Compared to the matched cohort, the Papa cohort had 34% fewer ED high-utilizers during the intervention year. Results provide preliminary evidence that a social companionship service may reduce readmissions and frequent ED use, and can inform future trials on companion-based interventions.

